# Red blood cell (RBC) transfusion rates among US chronic dialysis patients during changes to Medicare end-stage renal disease (ESRD) reimbursement systems and erythropoiesis stimulating agent (ESA) labels

**DOI:** 10.1186/1471-2369-15-116

**Published:** 2014-07-11

**Authors:** Katherine A Cappell, Sanatan Shreay, Zhun Cao, Helen V Varker, Carly J Paoli, Matthew Gitlin

**Affiliations:** 1 Truven Health Analytics, 777 East Eisenhower Parkway, Ann Arbor, MI 48108, USA; 2 Amgen, Thousand Oaks, CA, USA

**Keywords:** Transfusion, Chronic dialysis, Medicare prospective payment system, End-stage renal disease, Erythropoiesis stimulating agent

## Abstract

**Background:**

Several major ESRD-related regulatory and reimbursement changes were introduced in the United States in 2011. In several large, national datasets, these changes have been associated with decreases in erythropoiesis stimulating agent (ESA) utilization and hemoglobin concentrations in the ESRD population, as well as an increase in the use of red blood cell (RBC) transfusions in this population. Our objective was to examine the use of RBC transfusion before and after the regulatory and reimbursement changes implemented in 2011 in a prevalent population of chronic dialysis patients in a large national claims database.

**Methods:**

Patients in the *Truven Health MarketScan Commercial* and *Medicare Databases* with evidence of chronic dialysis were selected for the study. The proportion of chronic dialysis patients who received any RBC transfusion and RBC transfusion event rates per 100 patient-months were calculated in each month from January 1, 2007 to March 31, 2012. The results were analyzed overall and stratified by primary health insurance payer (commercial payer or Medicare).

**Results:**

Overall, the percent of chronic dialysis patients with RBC transfusion and RBC transfusion event rates per 100 patient-months increased between January 2007 and March 2012. When stratified by primary health insurance payer, it appears that the increase was driven by the primary Medicare insurance population. While the percent of patients with RBC transfusion and RBC transfusion event rates did not increase in the commercially insured population between 2007 and 2012 they did increase in the primary Medicare insurance population; the majority of the increase occurred in 2011 during the same time frame as the ESRD-related regulatory and reimbursement changes.

**Conclusions:**

The regulatory and reimbursement changes implemented in 2011 may have contributed to an increase in the use of RBC transfusions in chronic dialysis patients in the *MarketScan* dataset who were covered by Medicare plus Medicare supplemental insurance.

## Background

As of the end of 2010, the total treated end-stage renal disease (ESRD) population reached 594,374 patients in the United States [1]. Within this population, approximately 70% were treated with dialysis [1]. Anemia is a common complication among patients with ESRD [2] and erythropoiesis stimulating agents (ESAs) have been widely utilized for anemia management in this population over the past two decades [3,4]. Prior to the development of ESAs, the treatment options for anemia were limited to red blood cell (RBC) transfusions, and to a lesser extent, androgen and iron therapy [5]. In the pre-ESA era, 10-20% of dialysis patients received at least one RBC transfusion in a three month period to avoid severe anemia [6]. However, after the introduction of epoetin alfa in 1989, the use of RBC transfusion by Medicare-sponsored dialysis patients declined significantly to less than 5% with at least one RBC transfusion in a three month period [6]. The primary registration trials used for the approval of epoetin alfa demonstrated correction of anemia and virtual elimination of transfusions (>90% reduction) in patients treated with ESAs to a mean hemoglobin (Hb) of 11.7 g/dL (within the target range of 10.7 to 12.7 g/dL) [7]. From 1992 to 2000, the mean population Hb in prevalent dialysis patients rose from about 9.8 to 11.2 g/dL while the overall transfusion rate was halved [8].

Since the introduction of ESAs, several randomized trials have reported an increased risk of mortality and major cardiovascular events in pre-dialysis patients with CKD treated with ESAs [9-11]. The results of the two earlier trials prompted the US Food and Drug Administration (FDA) to issue a black box warning for all ESAs in March of 2007; the warning recommended that ESAs be used at the lowest level necessary to prevent RBC transfusions [12]. The later TREAT trial enrolled moderately anemic, pre-dialysis patients with type 2 diabetes and CKD and compared treatment with darbepoetin alfa to Hb of approximately 13 g/dL to placebo. Treatment with darbepoetin alfa was not found to reduce the risk of death or major cardiovascular or renal events and was associated with an increased risk of stroke [11]. As a result of the accumulation of studies demonstrating that ESA use is associated with increased risk of adverse events, in February 2010, the FDA released a drug safety communication stating that the manufacturers of all ESAs are required to implement a risk evaluation mitigation stragety (REMS). As part of the REMS, the FDA required that patients must be provided with a printed medication guide explaining the risks and benefits of ESAs each time the ESA is dispensed [13].

Several major ESRD-related regulatory and reimbursement changes were introduced in the United States in 2011. First, under the Medicare Prospective Payment System (PPS) for ESRD implemented by the Centers for Medicare & Medicaid Services (CMS) in January 2011, reimbursement for dialysis services was bundled to include formerly separately billable medications (i.e., ESAs, intravenous iron, active vitamin D, and ESRD-related antibiotics) [14]. Second, also in January 2011, a quality incentive program (QIP) was implemented by CMS to ensure that dialysis facilities maintain a high quality of care in the context of a cost-constrained payment system. The QIP focused on dialysis adequacy and avoidance of anemia (maintaining patients’ Hb < 12 g/dL and > 10 g/dL). In July 2011, CMS proposed removing the lower Hb measure (<10 g/dL); this revision was finalized in the November 2011 CMS Final Rule and effective on January 1, 2012 [15]. Finally, in June 2011, the ESA label information was revised, the primary changes being removal of the Hb target range of 10 to 12 g/dL and inclusion of dosing and administration language focused on reduction or interruption of ESA dosing when Hb concentrations approached or exceeded 11 g/dL [7,16].

Associated with the recent regulatory and reimbursement changes just described, ESA doses and Hb concentrations in dialysis patients in the US have decreased. According to the Dialysis Outcomes and Practice Patterns Study (DOPPS) Practice Monitor (DPM), from August 2010 to December 2012, the percentage of ESA-treated patients with a Hb concentration < 10 g/dL increased from 9% to 20% and those with a Hb concentration > 12 g/dL decreased from 26% to 9% [17]. In the Study to Evaluate the Prospective Payment System Impact on Small Dialysis Organizations (STEPPS), in a group of dialysis patients, it was found that the percent of patients with a monthly Hb concentration <10 g/dL increased from 12.7% to 16.8% and the percent of patients with a monthly Hb concentration >12 g/dL decreased from 28.7% to 18.5% between Q4 2010 and Q2 2011; during the same time period, the median cumulative monthly dose of intravenous epoetin alfa decreased by approximately 15% [18]. As well as the evidence demonstrating that Hb concentrations are declining in the US dialysis population, evidence from several sources suggests that RBC transfusion rates have increased in this population since the regulatory and reimbursement changes implemented 2011 [1,18-20].

The objective of the present study was to examine the proportion receiving RBC transfusion and RBC transfusion rates before and after the regulatory and reimbursement changes implemented in 2011 in a prevalent population of chronic dialysis patients. In order to obtain adequate information about the magnitude of year-to-year variation in the utilization of RBC transfusions prior to the regulatory and reimbursement changes, we examined data from 2007–2012. Previous reports of recent RBC transfusion rates have focused on patients with Medicare insurance [1,19]. This study adds to the current literature by examining RBC transfusions in a population of chronic dialysis patients aged 64 and under with employer-sponsored commercial insurance. We additionally examined RBC transfusions in a population of patients with Medicare insurance plus employer-sponsored Medicare supplemental insurance.

## Methods

### Data sources

This retrospective, observational cohort study was conducted using data from the *MarketScan® Commercial Claims and Encounters (Commercial) Database* and the *Medicare Supplemental and Coordination of Benefits (Medicare) Database* for the time period of January 1, 2007 to March 31, 2012. These databases are proprietary databases and are available for licensure for a fee. As the owner of the databases, Truven Health Analytics uses these data for analyses for commercial, government and academic clients. These databases are constructed from privately insured paid medical and prescription drug claims. The *Commercial Database* contains the health care experiences of privately insured individuals aged 64 and younger and covered under a variety of fee-for-service, fully capitated, and partially capitated health plans. It is constructed from claims and enrollment data provided by >130 large, employer-sponsored health plans across the United States. Over 37 million individuals are included in the *Commercial Database*, encompassing employees, their spouses, and their dependents. The *Medicare Database* contains the claims and enrollment data of approximately three million retirees with Medicare Supplemental insurance paid for by employers; both the Medicare-covered portion of payment and the employer-paid portion are included in the database. Both databases provide detailed cost, use, and outcomes data for healthcare services performed in both inpatient and outpatient settings as well as outpatient prescription drug claims. Medical claims are linked to prescription drug claims and enrollment data through unique enrollee identifiers. All database records are de-identified and fully compliant with US patient confidentiality requirements set forth in the Health Insurance Portability and Accountability Act (HIPAA).

For patients under 65 years of age, eligibility for Medicare coverage for ESRD-related services does not begin until the fourth month of hemodialysis treatment (or the first month for home dialysis with appropriate training). Additionally, after becoming eligible for Medicare, patients with existing employer-sponsored health insurance must undergo a 30-month “coordination period” during which time the employer is designated the primary payer and Medicare the secondary payer for healthcare services. It is only after this 30-month coordination period that Medicare assumes responsibility as the primary payer for healthcare services. In the MarketScan Databases, an individual does not appear in the Medicare database until the data-contributing employer indicates that the employee/dependent is eligible for Medicare coverage (i.e., Medicare coverage status is not independently verified). It is likely that employers do not indicate that an employee/dependent is eligible for Medicare until Medicare becomes the primary health insurance payer. Therefore, it is likely that the majority of patients who are eligible for Medicare coverage but still in the 30-month coordination period appear in the Commercial database.

### Study population

All patients in the *Commercial* and *Medicare Databases* with evidence of chronic dialysis between January 1, 2007 and March 31, 2012 were selected for the study. Chronic dialysis was defined as three consecutive calendar months containing at least one medical claim with a code specific to reimbursement for chronic dialysis or for chronic dialysis-related procedures and services (see Additional file 1 for chronic dialysis codes). For the base-case analysis, the index date was the date of the first dialysis claim that occurred in the first of the three consecutive months of dialysis claims. Patients were additionally required to have dialysis claims in at least 70 percent of the calendar months between the index date and the end of the follow-up period. The variable duration follow-up period ended with the earliest of the end of continuous health plan enrollment, the end of the study period (March 31, 2012), inpatient death^a^, or kidney transplant (see Additional file 2 for kidney transplant codes). These selection criteria are consistent with the methodology used to identify chronic dialysis patients by the United States Renal Data System (USRDS) [1]. In addition to the USRDS criteria, patients were required to be at least 18 years of age on the index date and to have at least 180 days of continuous enrollment before and 90 days of continuous enrollment after the index date. Consistent with Ibrahim and colleagues [8], patients were excluded from the analysis if they had evidence of cancer, a hematological condition, or a blood dyscrasia during the 180-day pre-index period (see Additional file 3 for codes). The purpose of these exclusions was to eliminate patients who may have received blood transfusions to treat conditions other than ESRD.

Two sensitivity analyses that evaluated the robustness of the base-case definition of the chronic dialysis population were performed; one used a different index date and a second used a different set of patient selection criteria. In the first sensitivity analysis (SA1), the patient selection criteria were identical to those used in the base-case analysis, except that the index date was the date of the first dialysis claim in the *third* of the three consecutive months of dialysis claims. In a second sensitivity analysis (SA2), an alternative method for identifying chronic dialysis patients consistent with Gitlin et al. was used [21]. Chronic dialysis patients were defined as patients at least 18 years old and with at least two medical claims with a chronic dialysis code (see Additional file 1 for chronic dialysis codes) at least 30 but no greater than 365 days apart. The set of dialysis codes used in SA2 was narrower than the set of codes used to identify dialysis in the main analysis and in SA1; specifically, the codes were the monthly capitation payment and composite rate codes specific to reimbursement for chronic dialysis.

### Outcomes

#### Patient characteristics

Health insurance enrollment information as of the index date was used to determine patients’ age, gender, geographic region of residence, residence in an urban area, health insurance plan type, and primary health insurance payer (commercial payer or Medicare). Dialysis modality (hemodialysis, peritoneal dialysis, or unknown) was also determined on the index dialysis claim (see Additional file 1 for dialysis type codes) as was the year of the index date. Inpatient and outpatient medical claims from the 180-day period prior to the index date were examined for the presence of International Classification of Disease, Ninth Revision, Clinical Modification (ICD-9-CM) diagnosis codes indicating specific co-morbid conditions. Claims incurred during the 180-day pre-index period were also used to calculate the Charlson comorbidity index (CCI) with Deyo modifications [22,23]. The number of hospitalizations and the total number of hospitalized days that occurred during the pre-index period were also recorded.

#### RBC transfusions

The proportion of chronic dialysis patients who received any RBC transfusion and RBC transfusion event rates per 100 patient-months were calculated in each month from January 1, 2007 to March 31, 2012. Only patients with health plan enrollment for the full month being reported were included in the calculations. Patients who transitioned from the Commercial Database to the Medicare Database during the study period contributed data to the commercial rates if enrolled in the Commercial Database for the full month and to the Medicare rates if enrolled in the Medicare Database for the full month. RBC transfusions were identified using ICD-9-CM diagnosis and procedure codes, Healthcare Common Procedure Coding System (HCPCS) codes, Current Procedure and Terminology (CPT-4) codes, and revenue codes (see Additional file 4 for RBC transfusion codes) on medical claims incurred during the follow-up period. Because some patients had multiple RBC transfusion claims within a short time frame and it was not possible to ascertain whether these claims were associated with a single RBC transfusion or multiple RBC transfusions that occurred in rapid succession, we combined individual transfusion claims within three days of one another into a transfusion episode, which was the unit of observation for this study (for details, see [21]). Monthly proportions of chronic dialysis patients receiving RBC transfusion and RBC transfusion event rates per 100 patient-months were measured overall and were stratified by primary health insurance payer (primary commercial or primary Medicare insurance).

### Statistical analyses

For patient baseline characteristics, means and standard deviations were computed for continuous variables and frequencies and percentages were calculated for categorical variables. Baseline characteristics were computed across all chronic dialysis patients who met the study inclusion criteria and also calculated separately for chronic dialysis patients with and without RBC transfusion during the follow-up period.

The proportion of patients receiving RBC transfusion as well as RBC transfusion event rates per 100 patient-months were estimated by month overall and stratified by payer and RBC transfusion setting. The monthly proportion of patients receiving RBC transfusion was calculated as the total number of patients receiving at least one RBC transfusion during the month divided by the number of chronic dialysis patients with health plan enrollment for the full month. The RBC transfusion rate per 100 patient-months was also assessed over each month and was calculated as the total number of RBC transfusion episodes during the month divided by the total person-days accumulated during the month multiplied by 30 and then by 100.

The formula used for the event rate calculation is shown below:

$$\begin{array}{ll} \mathrm{Transfusion}\;\mathrm{event}\;\mathrm{rat}e_{i}= & \frac{\mathrm{Total}\#\;\mathrm{of}\;\mathrm{Transfusion}\;\mathrm{Episode}s_{i}}{\mathrm{Total}\#\;\mathrm{Chronic}\;\mathrm{Dialysis}\;\mathrm{Patient}\;\mathrm{Day}s_{i}}\\ & \times 30\times 100 \end{array}$$

where *i* represents a specific month.

## Results

### Study sample

**Figure 1:**
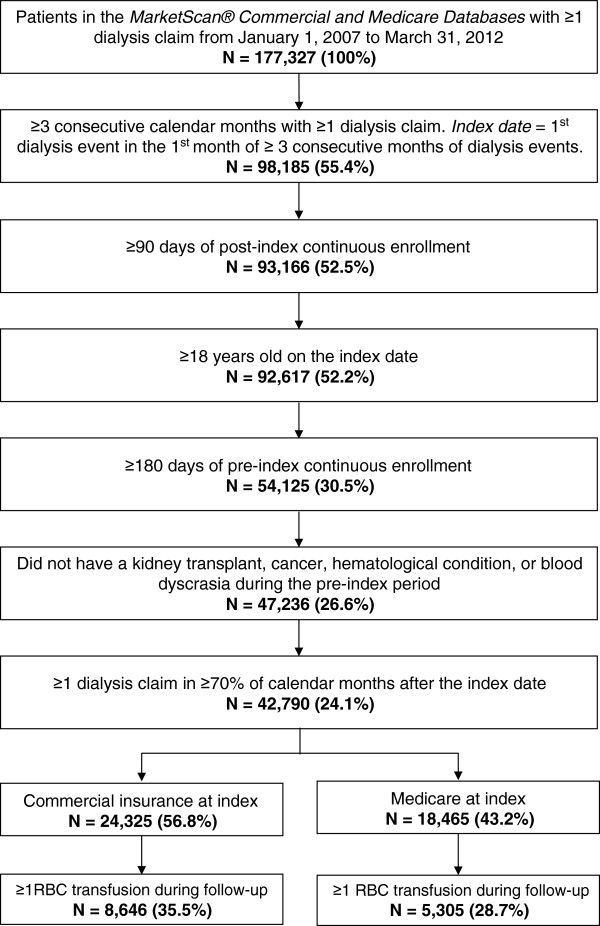
Selection of chronic dialysis patients, base-case analysis.

Between January 1, 2007 and March 31, 2012 there were 177,327 patients in the *MarketScan Commercial* and *Medicare Databases* with at least one dialysis claim (Figure 1). After application of the remaining criteria for inclusion in the base-case analysis, there were 42,790 individuals who qualified as chronic dialysis patients. In this sample of chronic dialysis patients, 24,325 (56.8%) had primary commercial insurance and 18,465 (43.2%) had primary Medicare insurance on the index date.^b^ Among the chronic dialysis patients with commercial insurance, 8,646 (35.5%) had at least one RBC transfusion during the variable duration follow-up period. In the sample of patients with primary Medicare insurance, 5,305 (28.7%) had at least one RBC transfusion during the follow-up period. As the outcome of interest in the present study is RBC transfusion, in the discussion that follows, we present the demographic and clinical characteristics of the transfused cohorts only.

The demographic, clinical, and treatment characteristics of chronic dialysis patients with at least one RBC transfusion who were included in the base-case analysis are shown in Additional files 5 and 6 stratified by payer on the index date (commercial: n = 8,646; Medicare: n = 5,305). The average age of patients was 52.1 years (SD = 10.0 years) in the primary commercial insurance sample and 74.7 years (SD = 7.5 years) in the primary Medicare insurance sample, consistent with the expectation that Medicare patients are mostly over age 65 while commercial patients are all under age 65. Four out of ten of the commercially insured patients (44.3%) and the Medicare patients (44.0%) were female.

As shown in Additional file 6, during the six-month pre-index period, the three most common comorbid conditions measured in both the primary commercial insurance and primary Medicare insurance chronic dialysis populations were hypertension (commercial: 66.5%, n = 5,753; Medicare: 66.7%, n = 3,536), diabetes (commercial: 51.9%%, n = 4,483; Medicare: 51.5%, n = 2,731), and coronary artery disease (commercial: 24.5%, n = 2,117; Medicare: 40.8%, n = 2,164). The Medicare insurance population had higher rates of most of the cardiovascular comorbidities measured (i.e., coronary artery disease, congestive heart failure, dysrhythmia, cerebrovascular accident/transient ischemic attack) than did the commercially insured population. The mean Charlson comorbidity score was 3.3 (SD = 2.2) in the primary commercial insurance population and 3.5 (SD = 2.0) in the primary Medicare insurance population. During the pre-index period, 38.3% (n = 3,314) of the primary commercial insurance sample and 39.4% (n = 2,090) of the primary Medicare insurance population experienced at least one hospitalization.

On the index date, approximately 70% of patients in both the primary commercial insurance and primary Medicare insurance samples received hemodialysis and the remaining patients received either peritoneal dialysis or dialysis via an unknown modality (Additional file 5). As shown in Additional file 6, the mean duration of follow-up was 645.5 days (SD = 476.9 days) for primary commercial insurance patients and 677.4 days (SD = 509.1 days) for primary Medicare insurance patients. During the follow-up period, 88.1% (n = 7,617) of primary commercial insurance patients and 86.4% (n = 4,585) of primary Medicare insurance patients had at least one inpatient transfusion and 25.7% (n = 2,223) of primary commercial insurance patients and 26.8% (n = 1,421) of primary Medicare insurance patients had at least one outpatient transfusion.

### Monthly proportions of chronic dialysis patients with RBC transfusion and RBC transfusion event rates

**Figure 2:**
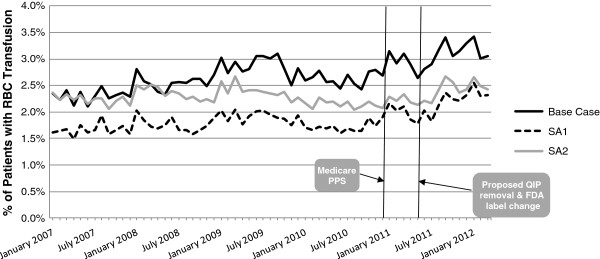
**Monthly percent of chronic dialysis patients with RBC transfusion using three definitions of chronic dialysis.** Detailed Legend: Base Case: USRDS definition of chronic dialysis, index date = month 1. SA1: sensitivity analysis #1; USRDS defintion of chronic dialysis, index date = month 3. SA2: sensitivity analysis #2; alternative definition of chronic dialysis [16].

Figure 2 depicts the proportions of patients with RBC transfusion by month across the three different methods (base-case, SA1, and SA2) used to define the chronic dialysis population. The overall proportions of patients with RBC transfusion differed in magnitude across the three methods; however, the trends over time were consistent among the three definitions. A similar pattern was found among the three different methods in the monthly RBC transfusion event rates per 100 patient-months (see Additional file 7). The remainder of this discussion will therefore focus on the results generated in the base-case population of chronic dialysis patients.

As depicted in Figure 2, in the base-case population overall, the proportion of patients with RBC transfusion increased from January 2007 to March 2012. The percent of patients with at least one RBC transfusion was 2.4% in January of 2007, 3.0% in January of 2009, 3.2% in January of 2011, and 3.4% in January of 2012. Similarly, the RBC transfusion event rate per 100 patient-months increased from 2.6 in January of 2007 to 3.2 in January of 2009 to 3.3 in January of 2011 and to 3.6 in January of 2012 (see Additional file 7).

**Figure 3:**
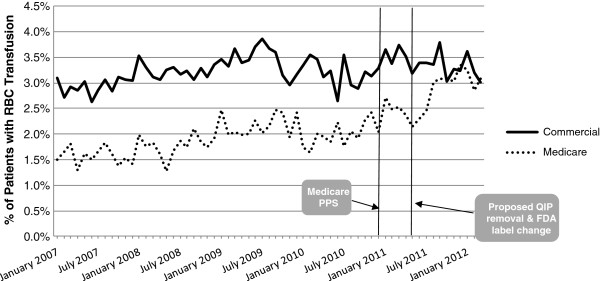
Monthly percent of chronic dialysis patients with RBC transfusion, base case population stratified by payer.

Figure 3 depicts the monthly proportions of patients with RBC transfusion separately for the primary commercial insurance and primary Medicare insurance populations (see Additional file 8 for the monthly RBC transfusion event rates per 100 patient-months stratified by primary insurance payer). In each year of the study, a higher proportion of the patients with primary commercial insurance than with primary Medicare insurance received RBC transfusion. Patients with primary commercial insurance also had higher transfusion event rates per 100 patient-months than those with primary Medicare insurance overall (see Additional file 8).

While the upward trend in the monthly proportions of patients with RBC transfusion and RBC transfusion event rates that was observed from January 2007 to March 2012 overall (Figure 2, Additional file 7) was pronounced in the primary Medicare insurance population the upward trend was not observed in the primary commercial insurance population (Figure 3, Additional file 8).

**Figure 4:**
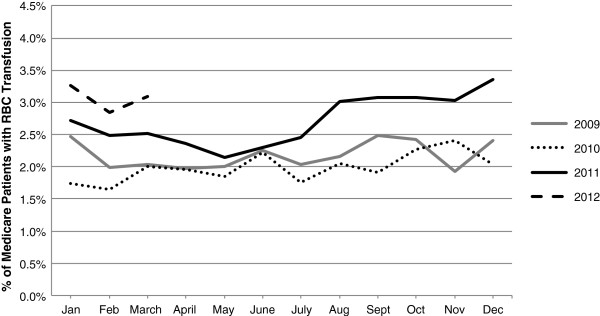
Percent of Medicare chronic dialysis patients with RBC transfusion, base case population stratified by year.

**Figure 5:**
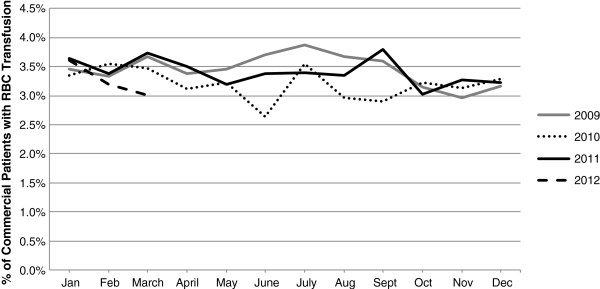
Percent of commercial chronic dialysis patients with RBC transfusion, base case population stratified by year.

Figures 4 and 5 depict the monthly proportions of patients with RBC transfusion separately for the primary commercial insurance and primary Medicare insurance populations stratified by calendar year (see Additional files 9 and 10 for the RBC transfusion event rates per 100 patient-months separately for the primary commercial insurance and primary Medicare insurance populations stratified by calendar year). In the primary Medicare insurance population, while the monthly proportions of patients with RBC transfusion and RBC transfusion event rates were similar in 2009 and 2010, the proportions and rates were higher in 2011, especially beginning in June-July 2011, (Figure 4, Additional file 9). This pattern of results was not evident in the primary commercial insurance population; rather, the proportion of patients with RBC transfusion and the RBC transfusion event rates remained consistent from 2009–2012 in this population (Figure 5, Additional file 10).

## Discussion

This retrospective study was conducted in a population of chronic dialysis patients with employer-sponsored health insurance or Medicare insurance with employer-sponsored Medicare supplemental insurance identified from the *Truven Health MarketScan Research Databases*. Consistent with the findings in the USRDS Medicare dataset [1,19], we found that the 2011 dialysis regulatory and reimbursement changes were associated with an increase in the proportion of chronic dialysis patients with RBC transfusion in the Medicare population. In the primary Medicare insurance population, while the monthly proportions of patients with RBC transfusion and RBC transfusion rates were similar in 2009 and 2010, the proportions and rates were higher in 2011, especially beginning in June-July 2011 (Figure 4, Additional file 9) suggesting that the increase in use of RBC transfusion in chronic dialysis patients may have been related to the regulatory and reimbursement changes implemented in 2011. This pattern of results was not evident in the primary commercial insurance population (Figure 5, Additional file 10); rather, the proportion of patients with RBC transfusion and the RBC transfusion event rates remained consistent from 2009–2012 in this population. The fact that the 2011 increase in RBC transfusion use was not evident in the commercially insured population suggests that the changes in RBC transfusion utilization may have been more directly related to the CMS regulatory changes, which applied only to the Medicare population, than to the ESA label changes, which applied to both the commercial and Medicare populations.

Our finding of higher transfusion rates in the commercially insured population as compared to the Medicare population was counterintuitive. One might expect that transfusion rates would be lower in a population of younger, healthier patients; the patient demographic and clinical characteristics presented in Additional files 5 and 6 indicate that the commercially insured patients were 22.6 years younger on average and had lower rates of most of the baseline comorbidities measured, especially the cardiovascular comorbidities. To the best of our knowledge, there are no other published data on RBC transfusion rates for primarily commercially insured chronic dialysis patients; therefore, we have no context in which to place the findings presented here. However, we have several hypotheses regarding our counterintuitive finding. First, while the commercially insured population may have appeared healthier than the Medicare population with respect to the comorbidities that were measured, it is possible that the commercially insured patients were in fact a less healthy population along dimensions that were not measured. Second, there may be systematic differences between the commercially and Medicare insured populations that impact the treatments that physicians are likely to recommend. For example, the Medicare insured patients had higher rates of cardiovascular comorbidities than their commercially insured counterparts; given the risk of adverse cardiovascular events (e.g., transfusion associated circulatory overload) associated with RBC transfusions, perhaps physicians are less likely to prescribe RBC transfusion to patients with cardiovascular comorbidities. Finally, it is possible that transfusion rates were underestimated in the Medicare sample for transfusions that took place during hospital re-admissions within 60 days after an inpatient admission. Medicare beneficiaries are only responsible for a Part A deductible for each benefit period. A benefit period begins on the admission date and ends when the beneficiary has not received any inpatient hospital care for 60 consecutive days. Any subsequent inpatient claims incurred before this time do not appear in the Medicare database because there is no patient responsibility for payment; therefore, re-admissions that occur within this 60 day window are not captured. Because the present study was not designed to measure or control for differences between the commercially insured and Medicare populations, we are unable to provide any definitive explanation for our counterintuitive finding; this question can be addressed in future research. However, because we are the first to report RBC transfusion rates in this population, we have begun to fill a gap in the literature.

In contrast, our findings in the Medicare population were consistent with previous reports. The post-January 2011 rise in the proportions of dialysis patients with RBC transfusion observed in the MarketScan primary Medicare insurance population has been observed in several other national datasets. In the USRDS dataset, which consists of a population of prevalent dialysis patients with Medicare insurance, while the transfusion rate per 100 patient months remained steady prior to the regulatory and reimbursement changes at approximately 2.5 throughout 2009 and most of 2010, they began to increase in October to November of 2010 and had increased to over 3.0 by November 2011 [19]. A 2011 rise in the proportion of chronic dialysis patients with at least one RBC transfusion per month was also observed in the population of patients with hospital-based transfusions identified the DOPPS population [20]. However, in the DOPPS population, the rise in the proportion of dialysis patients with transfusion occurred mainly between January 2011 (2.6%) and May 2011 (6.9%), whereas in the present MarketScan dataset, the rise in the primary Medicare insurance population occurred between May (2.1%) and December (3.4%) [24]. Finally, an increase in the proportion of patients with transfusion was also observed in the Study to Evaluate the Prospective Payment System Impact on Small Dialysis Organizations (STEPPS). The authors of this study reported that the proportion of prevalent dialysis patients with at least one transfusion in a calendar quarter increased from 1.8% in Q4 2010 to 2.9% in Q2 2011 [18].

An increase in the use of RBC transfusions in the chronic dialysis population may result in an associated increase in transfusion-related complications, such as hemolytic and non-hemolytic transfusion reactions, infections, transfusion-related acute lung injury, transfusion-associated circulatory overload, and hyperkalemia [25-28]. In previous work, we showed that while infrequent, payments for RBC transfusion complications were very high when complications occurred; mean payments for complications ranged from $213 (SD = $168) for delayed hemolytic transfusion reactions to $19,466 (SD = $15,424) for congestive heart failure [21]. Additionally, RBC transfusions can result in sensitization to human leukocyte antigen (HLA), which reduces the opportunity for future kidney transplantation [29]. However, the risk of adverse cardiovascular events associated with ESA treatment should also be considered when determining the appropriate anemia management strategy for chronic dialysis patients [9-11]. The potential for high-cost complications associated with RBC transfusion as well as the high cost of adverse events associated with ESA use should be considered by policy makers when determining the appropriate reimbursement policy for chronic dialysis patients. At present, the hemoglobin level at which the risk of adverse events in anemic CKD patients (with or without dialysis) treated with ESAs increases is unknown [30]. This is an important gap in knowledge that, when addressed, will better enable clinicians and policy makers to weigh the costs and benefits of the various anemia management options.

Our study had several limitations. Patients in our sample had either commercial insurance or commercial plus Medicare supplemental insurance as their primary coverage and therefore, these results may not be generalizable to patients who are uninsured, are covered only by Medicare, or have other types of insurance coverage. Patient clinical characteristics, dialysis services, and RBC transfusions were identified using ICD-9-CM, HCPCS, CPT, and revenue codes as recorded on administrative claims. It is possible that incomplete, missing, or miscoded claims impacted the study findings; however, coding errors are likely equally distributed both across time and across the commercially insured and primary Medicare insurance cohorts. Inclusion in the commercially insured and primary Medicare insurance samples required at least nine months (180 pre- and 90 days post-index) of continuous health plan enrollment. This requirement may have biased the sample toward a healthier-than-usual subset of the overall chronic dialysis population. For each patient, all RBC transfusion claims identified within a three day period after an initial transfusion claim were combined and defined as a single “transfusion episode”; use of this methodology may have resulted in an underestimation of the transfusion rates. These analyses included all RBC transfusions and did not distinguish between those administered for treatment of chronic anemia and those administered in the context of an acute, blood-loss related event. As discussed above, it is possible that transfusion rates were underestimated in the Medicare sample for transfusions that took place during hospital re-admissions within 60 days after an inpatient admission. Information about Hb levels and ESA utilization was not available for this population; therefore, we were unable to make direct associations between these measures and use of RBC transfusions. We did not measure dialysis vintage, severity of CKD, or anemia status, and therefore cannot identify potential differences between the commercially- and Medicare-insured populations that could potentially help to explain the differences that we found in the RBC transfusion rates in these two populations. Finally, we observed an association between the introduction of regulatory and reimbursement changes and an increase in RBC transfusion rates in 2011 for patients with Medicare supplemental insurance, but we did not observe the same association for patients with commercial insurance. It is out of the scope of this study to determine if the difference in transfusion rates between the Medicare and commercially insured populations is because the regulatory and reimbursement change mainly affects Medicare, or because Medicare patients had a lower rate to start with due to their older age (i.e., Medicare patients were 23 years older than commercial patients in our study), or both. Future studies will be needed to help address this question.

## Conclusions

The regulatory and reimbursement changes implemented in 2011 may have contributed to an increase in the use of RBC transfusion in patients covered by Medicare supplemental insurance. No increase in RBC transfusion was found in the commercially insured population. Future research is needed to explain this difference in the Medicare and Commercial populations. However, the fact that the 2011 increase in RBC transfusion use observed in the primary Medicare insurance population was not evident in the commercially insured population suggests that the changes in RBC transfusion utilization may have been associated with the CMS regulatory changes, which applied only to the Medicare population, and not the ESA label changes, which applied to both the commercial and Medicare populations.

### Endnotes

^a^Using administrative claims data, death is only observable when recorded as a discharge status on an inpatient claim. If death occurs outside of the inpatient setting, it will manifest in the patient’s data as the end of continuous enrollment.

^b^For SA1, 39,387 patients met the study inclusion criteria; 22,340 patients had commercial insurance (56.7%) and 17,047 (43.3%) had Medicare supplemental insurance on the index date. For SA2, 99,202 patients met the study inclusion criteria; 57,361 patients had commercial insurance (57.8%) and 41,841 (42.2%) had Medicare supplemental insurance on the index date.

## Abbreviations

CCI: Charlson comorbidity index; CMS: Centers for Medicare & Medicaid Services; CPT: Current Procedure and Terminology; CR: Composite rate; dL: Deciliter; DOPPS: Dialysis Outcomes and Practice Patterns Study; DPM: DOPPS Practice Monitor; ESA: Erythropoiesis stimulating agent; ESRD: End-stage renal disease; Hb: Hemoglobin; HLA: Human leukocyte antigen; HCPCS: Healthcare Common Procedure Coding System; ICD-9-CM: International Classification of Disease, Ninth Revision, Clinical Modification; MCP: Monthly capitation payment; PPS: Prospective payment system; QIP: Quality incentive program; RBC: Red blood cell; SA1: Sensitivity analysis #1; SA2: Sensitivity analysis #2; SD: Standard deviation; STEPPS: Study to Evaluate the Prospective Payment System Impact on Small Dialysis Organizations; USRDS: United States Renal Data System.

## Competing interests

This study was funded by Amgen Inc. Katherine Cappell and Helen Varker are employees of Truven Health Analytics, who was paid by Amgen in connection with the development of this manuscript. Zhun Cao was an employee of Truven Health Analytics when this study was conducted. Carly Paoli is an employee of and stockholder at Amgen Inc. Sanatan Shreay and Matthew Gitlin were employees of and stockholders at Amgen Inc. when this study was conducted.

## Authors’ contributions

KAC and ZC contributed to the study design, acquisition of the data, analysis and interpretation of the data, contributed to the writing and revision of the manuscript, and approved the final draft for submission. SS, CJP and MG contributed to the conception of the study, study design, analysis and interpretation of the data, contributed to the writing and revision of the manuscript, and approved the final draft for submission. HVV contributed to the study design, acquisition of the data, and approved the final draft for submission.

## Pre-publication history

The pre-publication history for this paper can be accessed here:

http://www.biomedcentral.com/1471-2369/15/116/prepub

## Supplementary Material

Additional file 1 **Codes used to identify chronic dialysis and dialysis type.** Description of data: Diagnosis, procedure, and revenue codes used to identify the presence of chronic dialysis and the type of chronic dialysis (hemodialysis, peritoneal dialysis, or unknown dialysis type). Click here for file

Additional file 2 **Codes used to identify kidney transplant.** Description of data: Procedure codes used to identify the presence of kidney transplant. Click here for file

Additional file 3 **Codes used to identify cancer, hematological conditions, and blood dyscrasias.** Description of data: Diagnosis codes used to identify the presence of cancer, hematological conditions, and blood dyscrasias). Click here for file

Additional file 4 **Codes used to identify RBC transfusions.** Description of data: Diagnosis, procedure, and revenue codes used to identify the presence of RBC transfusions. Click here for file

Additional file 5 **Patient demographic characteristics, transfused patients in base-case population.** Description of data: Demographic characteristic of patients with RBC transfusion in the base-case population. Click here for file

Additional file 6 **Patient clinical characteristics, transfused patients in base-case population.** Description of data: Clinical characteristic of patients with RBC transfusion in the base-case population. Click here for file

Additional file 7 **Monthly RBC transfusion event rate per 100 patient-months using three definitions of chronic dialysis.** Description of data: Monthly RBC transfusion event rate per 100 patient-months using three definitions of chronic dialysis. Click here for file

Additional file 8 **Monthly RBC transfusion event rates per 100 patient-months, base case population stratified by health insurance payer.** Description of data: Monthly RBC transfusion event rates per 100 patient-months, base case population stratified by health insurance payer. Click here for file

Additional file 9 **RBC transfusion event rate per 100 Medicare patient-months, base case population stratified by calendar year.** Description of data: RBC transfusion event rate per 100 Medicare patient-months, base case population stratified by calendar year. Click here for file

Additional file 10 **RBC transfusion event rate per 100 commercial patient-months, base case population stratified by calendar year.** Description of data: RBC transfusion event rate per 100 commercial patient-months, base case population stratified by calendar year. Click here for file
