# Supplementary material for: Red blood cell (RBC) transfusion rates among US chronic dialysis patients during changes to Medicare end-stage renal disease (ESRD) reimbursement systems and erythropoiesis stimulating agent (ESA) labels
Source: BMC Nephrol. 2014 Jul 11;15:116. doi: 10.1186/1471-2369-15-116 (PMC4112651; doi:10.1186/1471-2369-15-116)
Supplement: Additional file 7 — Monthly RBC transfusion event rate per 100 patient-months using three definitions of chronic dialysis. Description of data: Monthly RBC transfusion event rate per 100 patient-months using three definitions of chronic dialysis. [file 1471-2369-15-116-S7.doc]

Additional File 7. Monthly RBC transfusion event rate per 100 patient-months using three definitions of chronic dialysis

Base Case: USRDS definition of chronic dialysis, index date = month 1

SA1: sensitivity analysis #1; USRDS defintion of chronic dialysis, index date = month 3

SA2: sensitivity analysis #2; alternative definition of chronic dialysis [16]
