# Supplementary material for: Red blood cell (RBC) transfusion rates among US chronic dialysis patients during changes to Medicare end-stage renal disease (ESRD) reimbursement systems and erythropoiesis stimulating agent (ESA) labels
Source: BMC Nephrol. 2014 Jul 11;15:116. doi: 10.1186/1471-2369-15-116 (PMC4112651; doi:10.1186/1471-2369-15-116)
Supplement: Additional file 10 — RBC transfusion event rate per 100 commercial patient-months, base case population stratified by calendar year. Description of data: RBC transfusion event rate per 100 commercial patient-months, base case population stratified by calendar year. [file 1471-2369-15-116-S10.doc]

Additional File 10. RBC transfusion event rate per 100 commercial patient-months, base case population stratified by calendar year
